# Supplementary material for: Effects of an EPS Biosynthesis Gene Cluster of Paenibacillus polymyxa WLY78 on Biofilm Formation and Nitrogen Fixation under Aerobic Conditions
Source: Microorganisms. 2021 Jan 30;9(2):289. doi: 10.3390/microorganisms9020289 (PMC7911366; doi:10.3390/microorganisms9020289)
Supplement: Supplementary file 1 [file microorganisms-09-00289-s001.pdf]

Supplementary materials:

Table S1. Bacterial strains and plasmids used in this study.

Strain or plasmid	Description	Source
	Paenibacillus polymyxa	
WLY78	Wild-type strain	Laboratory stock
Δpep-1	*pep-1* in-frame deletion mutant	This study
Δpep-2	*pep-2* in-frame deletion mutant	This study
	E. coli	
JM109	General cloning host; *recA1, endA1, gyrA96, thi-1, hsdR17, supE44, relA1, Δ(lac-proAB)*/F'[*traD36, proAB+, lacIq, lacZ*ΔM15]	Sangon Biotech Co.
	Plasmids	
pRN5101	Temperature-sensitive *E. coli-Bacillus* shuttle vector, *Ery*^{R}	Ding yan qin's Lab
pRDpep-1	*pep-1* deletion vector based on pRN5101	This study
pRDpep-2	*pep-2* deletion vector based on pRN5101	This study

Table S2. Primers used in this study.

Gene name/ Location	Forward primer (5'–3')	Reverse primer (5'–3')	Target
	Primers for gene knockout and validation		
Upstream of *pep-1*	pep-1-up-F: CGGCCACGATGCGTCCGGCGTAGAGGGCTTACCTAAGGCGATATG	pep-1-up-R: GTGCAGTTGAAAGGTAAATCCGTAAAGGGAG	In-frame deletion of *pep-1*
Downstream of *pep-1*	pep-1-down-F: TTTACGGATTTACCTTTCAACTGCACAAGACC	pep-1-down-F: GACTGCGCAAAAGACATAATCGATAACGACAACATTGCAGGTG	In-frame deletion of *pep-1*
Upstream of *pep-2*	*pep-2*-up-F: CGGCCACGATGCGTCCGGCGTAGAGGACAGTATACGAATTGGGC	*pep-2*-up-R: GATTTTTGTGGAGCCCGTCACCAATTTATCTC	In-frame deletion of *pep-2*
Downstream of *pep-2*	*pep-2*-down-F: AATTGGTGACGGGCTCCACAAAAATCCTCCC	*pep-2*-down-R GACTGCGCAAAAGACATAATCGATACAGATCCGATTGTCCATTC	In-frame deletion of *pep-2*
	RT-PCR primers used for determination of the organization of the pep-1 and pep-2 gene clusters		
pepM *manC*	*pepM*-F: GCGTCCGAATTAACA	*manC*-R: CCCGTCACCAATTTATCTC	RT-PCR (Marked 1)*
pepN *pepM*	*pepN*-F: GGTTCGTGGACGCATTCA	*pepM*-R: AGCTCCGACTCCTCCTTCTGT	RT-PCR (Marked 2)*
manC *ugdH1*	*manC*-F: TACGGAGGCGAAGAAAGCA	*ugdH1*-R: TGAAAAAGGGGTTCGC	RT-PCR (Marked 3)*
GM004728 *pepN*	4728-F: GACCAGCTTGAACTGTCCGATGAG	*pepN*-R: ACGACAACATTGCAGGTG	RT-PCR (Marked 4)*
ugdH1 *pepJ*	*ugdH1*-F: TACCGCATCAGCCAAATCC	*pepJ*-R: TGAAGGGTTGCTGCGTGT	RT-PCR (Marked 5)*
pepJ *pepH*	*pepJ*-F: GAACACCTCTTCCACCTG	*pepH*-R: CAACCTTTCTGGCTGCGA	RT-PCR (Marked 6)*
pepH *pepF*	*pepH*-F： TGGTCCCTCCACTGATAGCA	*pepF*-R: GGAGCCTAAACGATTGTGG	RT-PCR (Marked 7)*
pepF *pepE*	*pepF*-F: GCTTCCGCTTGAACATCTC	*pepE*-R: GGTCTGGGCATTGGTGAT	RT-PCR (Marked 8)*

pepE *pepC*	*pepE*-F: GATAAACCGACGAACGCC	*pepC*-R: GTAGGCTTGATCGTCTT-GCTTCCG	RT-PCR (Marked 9)*
pepC *pepA*	*pepC*-F: CCGCTCAC-TTCATTCAGCTCCATC	*pepA*-R: GGTCTTGGACATGCGG-TATGGTG	RT-PCR (Marked 10)*
	qRT-PCR primers used for determination of *nif* gene expression		
nifH	*nifH*-F: AC-CTGCCAGCTCTTCATACTC	*nifH*-R: AACAGCCGGAATAC-GGACC	qRT-PCR
nifD	*nifD*-F: TCATTCCTGTAC-GCTGTGAGG	*nifD*-R: CACCGCCGA-TATTGTAGTCTC	qRT-PCR
nifK	nifK-F: GCGGA-GATGATTGCGGTATG	nifK-R: GGCGTCATAGCCTG-TAATATGTG	qRT-PCR

* The primers are used in the numbering areas of Figure 2A

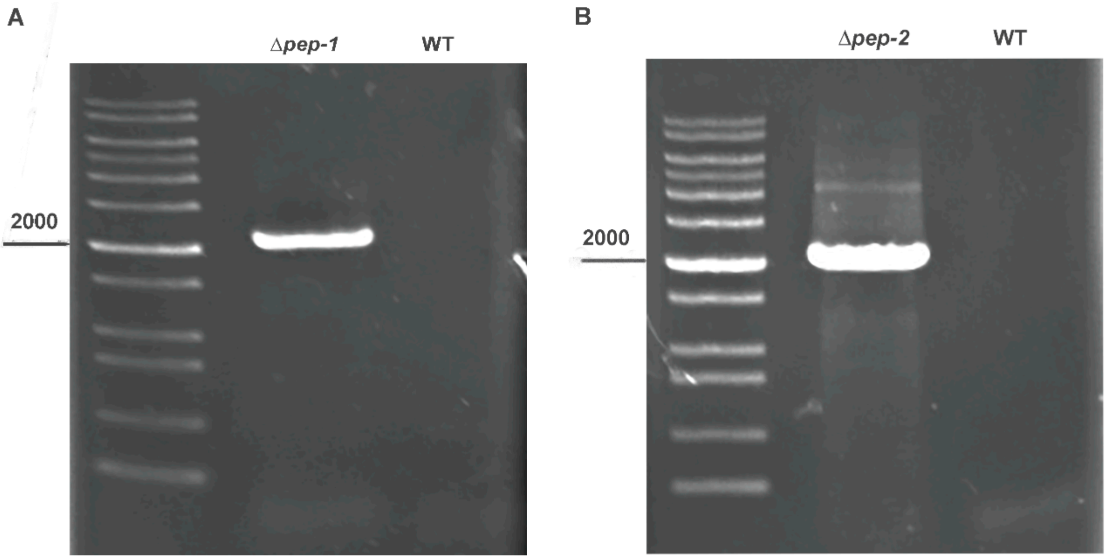

Figure S1. Identification of the knockout mutants (Δ*pep-1* and Δ*pep-2*) by PCR. (A) Identification of the Δ*pep-1* mutant. A 2072 bp DNA of fragment was obtained in Δ*pep-1* mutant by PCR with primers pep-1-up-F and pep-1-down-R, but it was not observed in *P. polymyxa* WLY78. The special 2072 bp DNA fragment in Δ*pep-1* mutant was produced in construction of this mutant by homologous recombination. For knockout of the *pep-1* cluster, about 1 kb upstream fragment flanking *ugdH2* was obtained by PCR with primers pep-1-up-F and pep-1-up-R, and 1 kb downstream fragment flanking *pepO* was obtained by PCR with primers pep-1-down-F and pep-1-down-R. Then, the two PCR fragments were used to make homologous recombination to get the Δ*pep-1* mutant in which the 11 genes (*pepO-ugdH2*) with a length of 12.6 kb DNA were removed and the ~2 kb DNA fragment (about 1 kb upstream fragment and 1 kb downstream fragment) remained. (B) Identification of the Δ*pep-2* mutant. A 2102 bp DNA fragment was obtained in the Δ*pep-2* mutant by PCR with primers pep-2-up-F and pep-2-down-R. Then these PCR fragments were sequenced. The special 2102 bp DNA fragment in Δ*pep-2* mutant was produced in construction of this mutant by homologous recombination. Similarily, for disruption of the *pep-2* cluster, about 1 kb upstream fragment flanking *manC* was obtained by PCR with primers pep-2-up-F and pep-2-up-R and 1 kb downstream fragment flanking *pepA* was obtained by PCR with primers pep-2-down-F and pep-2-down-R from the genomic DNA of *P. polymyxa* WLY78. The two PCR fragments were used to make homologous recombination to get the Δ*pep-2* mutant in which the 15 genes (*pepA-manC*) with a length of 16.5 kb DNA were removed and the ~2 kb DNA fragment (about 1 kb upstream fragment and 1 kb downstream fragment) remained.

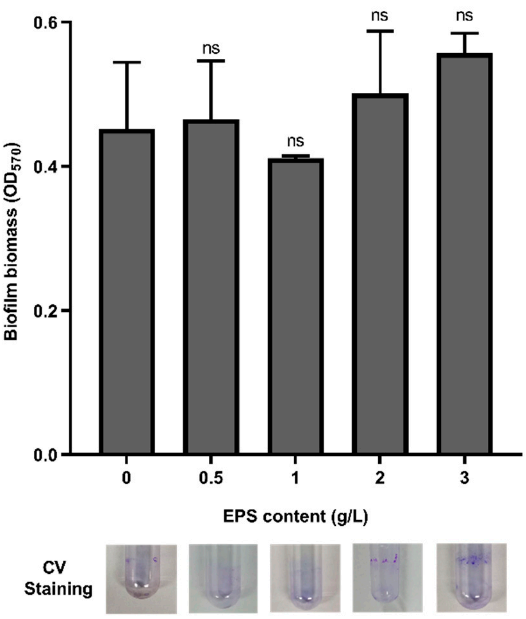

Figure S2. Impacts of exopolysaccharides (EPS) purified from *P. polymyxa* WLY78 on biofilm formation of the *pep-2* mutant. EPS was purified from *P. polymyxa* WLY78 and then different amounts (0-3 g/L) of the purified EPS were added to the liquid nitrogen-free medium supplemented with 20 mM NH_4Cl. After cultivation for 96 h, biofilm formation was assayed by CV staining. The results show that the purified EPS from *P. polymyxa* WLY78 could not obviuosly enhance the biofilm biomass. Results are representative of at least three independent experiments. Error bars indicate SD. Ns indicates nonsignificant.
